# Prognostic value of myocardial salvage index assessed by cardiovascular magnetic resonance in reperfused ST-segment elevation myocardial infarction

**DOI:** 10.3389/fcvm.2022.933733

**Published:** 2022-08-16

**Authors:** Shiru Zhang, Quanmei Ma, Yundi Jiao, Jiake Wu, Tongtong Yu, Yang Hou, Zhijun Sun, Liqiang Zheng, Zhaoqing Sun

**Affiliations:** ^1^Department of Cardiology, Shengjing Hospital of China Medical University, Shenyang, China; ^2^Department of Radiology, Guangdong Provincial People’s Hospital, Guangdong Academy of Medical Sciences, Guangzhou, China; ^3^Department of Radiology, Ganzhou Municipal Hospital, Ganzhou, China; ^4^Department of Cardiology, The First Affiliated Hospital of China Medical University, Shenyang, China; ^5^Department of Radiology, Shengjing Hospital of China Medical University, Shenyang, China; ^6^School of Public Health, Shanghai Jiao Tong University School of Medicine, Shanghai, China

**Keywords:** myocardial salvage index, cardiovascular magnetic resonance, ST-segment elevation myocardial infarction, major adverse cardiovascular event, prognosis

## Abstract

**Aims:**

Cardiovascular magnetic resonance (CMR) is a powerful tool to quantify the myocardial area at risk (AAR) and infarct size (IS), and evaluate the extent of myocardial salvage in acute ST-segment elevation myocardial infarction (STEMI). This study aimed to assess the prognostic value of myocardial salvage index (MSI) assessed by CMR in reperfused STEMI and investigate whether MSI could improve the predictive efficacy of the Global Registry of Acute Coronary Events (GRACE) risk score.

**Methods and results:**

About 104 consecutive patients who were hospitalized with first-time STEMI and received reperfusion therapy were prospectively enrolled. The primary endpoint was the incident of major adverse cardiovascular event (MACE) including all-cause mortality, non-fatal myocardial reinfarction and congestive heart failure within 36 months after the index event. Cox regression analysis was used to evaluate the prognostic association of MSI with MACE risk. About 21 (20.2%) patients developed MACE during the 3-year follow-up period, and patients with MSI < median had a higher incidence of MACE than those with MSI ≥ median [16 (30.8%) vs. 5 (9.6%), *P* = 0.007]. After adjusting all the parameters associated with MACE in univariate Cox analysis, MSI assessed by CMR remained independently significant as a predictor of MACE in multivariate Cox analysis (hazard ratio 0.963, 95% CI: 0.943–0.983; *P* < 0.001). Adding MSI to the GRACE risk score significantly increased the prognostic accuracy of the GRACE risk score (area under the curve: 0.833 vs. 0.773; *P* = 0.044), with a net reclassification improvement of 0.635 (*P* = 0.009) and an integrated discrimination improvement of 0.101 (*P* = 0.002).

**Conclusion:**

This study confirmed that MSI assessed by CMR had a good long-term prognostic value in reperfused STEMI and improve the prognostic performance of the GRACE risk score.

## Introduction

ST-segment elevation myocardial infarction (STEMI) is a common acute presentation of acute coronary syndromes (ACS), accounting for about 30% of ACS (1, 2). It is a main cause of morbidity and mortality worldwide and causes higher disease burden and risk in developing countries (3–5). As the current recommended treatment strategy for STEMI, primary percutaneous coronary intervention (P-PCI) can restore the patency of infarct-related arteries and limit the extent of irreversible myocardial injury, thereby improving the survival rate of patients (4, 6, 7). Although timely and effective reperfusion therapy could significantly reduce the mortality, the incidence of cardiovascular adverse events post-STEMI remains significant (7, 8). Salvaging damaged myocardium is the primary objective of reperfusion therapy in STEMI (9). From a clinical perspective, it is vital to assess myocardial salvage defined as the difference between the area at risk (AAR) and infarct size (IS) (10, 11). Because significant salvage leads to the long-term reconstruction of myocardial contractile function and improves the prognosis of patients (12, 13).

Cardiovascular magnetic resonance (CMR), as a good non-invasive imaging technique, is currently considered the gold standard to quantify myocardial damage following myocardial infarction, with high accuracy and reproducibility (14, 15). It has high spatial resolution and can be used to retrospectively evaluate the myocardial injury and myocardial salvage within 1 week after acute coronary artery occlusion. CMR imaging with its unique ability to provide a comprehensive determination of myocardial function and structure, including quantification of myocardial salvage, has become the choice to evaluate the survival of patients with acute STEMI (16, 17). Although using CMR to measure therapy efficiency has been proposed as part of risk assessment for STEMI patients, it has not been adopted widely (7). The Global Registry of Acute Coronary Events (GRACE) risk score remains the most accepted tool for risk stratification to identifying high-risk patients with adverse clinical outcomes (18).

This study aimed to (a) assess the prognostic value of myocardial salvage index (MSI) in patients with STEMI after P-PCI, and (b) investigate whether MSI assessed by CMR can improve the predictive efficacy of the GRACE risk score.

## Materials and methods

### Patient population

From November 2016 to January 2019, consecutive patients who were hospitalized with STEMI at our hospital and received reperfusion therapy by P-PCI were enrolled in this prospective study. STEMI patients treated with P-PCI should meet the following diagnostic criteria: (a) the presence of chest pain < 12 h from onset of pain to time of catheterization and (b) significant ST-segment elevation (at least 0.1 mV in ≥ 2 standard leads or at least 0.2 mV in ≥ 2 contiguous precordial leads) or a new left bundle branch block. P-PCI was urgently underwent based on the guidelines (7, 19). Exclusion criteria were as follows: (a) previous myocardial infarction or revascularization, (b) missing or poor quality of CMR data, (c) loss to follow up, and (d) contraindications for CMR or gadolinium-based contrast at the beginning of the study. About 104 patients fulfilled the criteria, gave written informed consent, underwent CMR examinations, and were eventually included. Clinical data pertaining to the demographic variables, lifestyle factors and medical history, laboratory parameters, CMR parameters and operational data of all patients were obtained by the trained researchers through medical records, imaging data, and surgical records. The Shengjing Hospital of China Medical University Ethics Committee approved this prospective study (2016PS373K), which followed the principles of the Helsinki Declaration.

### Cardiovascular magnetic resonance protocol and analysis

Patients, underdoing CMR at 3–7 days after P-PCI, were examined with a 3.0-T MR scanner (Philips Intera, Best, the Netherlands) using a 32-channel phased-array receiver coil after P-PCI. Data were evaluated and analyzed separately by two radiologists with 8 and 12 years of cardiothoracic experience, using the CVI software (version 5.9.1, Circle Cardiovascular Imaging Inc.). In order to assess the left ventricular function, a balanced turbo field echo-breath hold sequence was used to obtain two-chamber, four-chamber, and left ventricular short-axis cine images. The short-axis images of the left ventricular covered the entire left ventricle. T2-weighted images were obtained using the spectral attenuated inversion recovery sequence to evaluate for myocardial edema. Late gadolinium enhancement (LGE) scan was performed 10 min after the first intravenous injection of 0.2 mmol/kg gadolinium-based contrast agent with an inversion recovery technique. The phase-sensitive inversion recovery sequence was used to cover the entire left ventricle on the short axis. The operators were not aware of patients’ relevant clinical baseline information and the results of P-PCI.

The cardiac function indicators such as left ventricular ejection fraction (LVEF), left ventricular end-diastolic volume (LVEDV), and left ventricular end-systolic volume (LVESV) were analyzed by cine images. Myocardium whose signal intensity (SI) is greater than the mean SI of the distal myocardium by more than 2 standard deviations (SDs) in T2-weighted images was considered AAR (20), and infarcted myocardium was defined as the region of myocardium where the SI in LGE was greater than that of the remote myocardial region of interest by a threshold of 5 SDs (21). The AAR and IS were expressed in grams and as a percentage of the total left ventricular (LV) mass. Microvascular obstruction (MVO) was defined as the area of hypointense signal within an LGE area. Finally, the formula for calculating MSI is as follows: (AAR-IS)/AAR × 100.

### Clinical endpoints

The primary endpoint was the incident MACE, which was described as a composite endpoint that included non-fatal myocardial reinfarction, congestive heart failure, and all-cause mortality. Each component of the primary endpoint was considered a secondary endpoint. In the secondary endpoint analysis, if a participant had different types of events, the first event of each type was considered the endpoint event. Clinical follow-up was scheduled *via* outpatient visits, telephone interviews, or/and review of patients’ medical or hospitalization records at 1 week, 1, 3, 6, 12 months, and every 1 year thereafter of P-PCI. During the follow-up period, the interviewers were blinded to the patients’ clinical information and CMR data. Non-fatal myocardial reinfarction was defined as a transient increase in laboratory markers (troponin-I) specific to myocardial necrosis in combination with ischemic symptoms and/or typical electrocardiographic signs (development of pathologic Q-waves or ST-segment elevation or depression). Congestive heart failure was defined as episodes of cardiac decompensation (edemas, rales, dyspnea New York Heart Association class III-IV) requiring medical attention. All-cause mortality was assessed using standardized definitions (22). If more than one event occurred in a patient during the follow-up period, the first event was considered the primary endpoint for the MACE analysis.

### Statistical analysis

A χ^2^-test or Fisher’s exact test, when appropriate, was used to assess categorical variables, which are presented as numbers (percentage). The Kolmogorov-Smirnov test was used to examine the normality of data. Normally distributed continuous variables were presented as the mean ± *SD*, and a *t*-test was used for comparison between groups. Continuous variables with non-normally distribution were expressed as the medians with interquartile ranges (IQRs), and a non-parametric Mann–Whitney’s *U*-test was used for comparison between groups. The intra- and interobserver variability’s for reproducibility were assessed using the two-way mixed-effects intraclass correlation coefficient (ICC) and Bland-Altman plots. The cumulative incidence of primary endpoint and secondary endpoint between the two groups was evaluated using Kaplan-Meier curves, and the difference was compared by the log-rank test. Univariate Cox proportional-hazards regression was used to describe and analyze the longitudinal associations between variables in Tables 1, 2 and the clinical outcomes, hazard ratios (HR), and 95% confidence intervals (CI) were calculated. All associated variables in Tables 1, 2, which were with a *P*-value < 0.05 in univariate Cox proportional-hazards regression, were tested in the stepwise multivariate Cox analysis. The proportional-hazards assumption test based on Schoenfeld residuals was visually confirmed. The receiver operating characteristic (ROC) curves were generated, and areas under the curves (AUC) were calculated to compare the discriminatory performance of MSI, GRACE risk score, and the addition of MSI to GRACE risk score in predicting clinical outcomes using the DeLong method through the MedCalc Statistical Software version 15.2.2 (MedCalc Software Ltd., Ostend, Belgium). The best cut-off value of MSI for the prediction MACE was determined according to Youden’s index. And, incremental predictive ability from adding MSI to GRACE risk score was further analyzed using the net reclassification improvement (NRI) and the integrated discrimination improvement (IDI) with the SAS software, version 9.4 (SAS Institute Inc., Cary, North Carolina, United States). The initial data analysis was performed using the IBM SPSS version 26.0 (SPSS Inc., Chicago, IL, United States), and a two-sided *P*-value < 0.05 was considered statistically significant.

**TABLE 1:** Main patient characteristics.

	MSI ≥ Median MSI (*n* = 52)	MSI < Median MSI (*n* = 52)	*P*-value
Age, y	57.5 (46.3, 62.0)	61.0 (48.0, 65.8)	0.097
Male sex	47 (90.4)	38 (73.1)	0.022
**Cardiovascular risk factors**			
Current smoking	36 (69.2)	38 (73.1)	0.665
Hypertension	23 (44.2)	17 (32.7)	0.227
Diabetes mellitus	16 (30.8)	11 (21.2)	0.263
Anterior myocardial infarction	18 (34.6)	26 (50.0)	0.112
**Culprit lesion**			
Left main	0 (0.0)	1 (1.9)	0.134
Left anterior descending artery	18 (34.6)	24 (46.2)	
Left circumflex artery	5 (9.6)	9 (17.3)	
Right coronary artery	29 (55.8)	18 (34.6)	
**Killip class on admission**			
1	48 (92.3)	45 (86.5)	0.473
2	4 (7.7)	6 (11.5)	
3	0 (0.0)	0 (0.0)	
4	0 (0.0)	1 (1.9)	
Door-to-balloon time, min	87.5 (70.3, 131.0)	104.5 (73.3, 159.0)	0.261
**TIMI flow grade before PCI**			
0	42 (80.8)	41 (78.8)	0.750
1	2 (3.8)	3 (5.8)	
2	3 (5.8)	5 (9.6)	
3	5 (9.6)	3 (5.8)	
**TIMI flow grade after PCI**			
2	1 (1.9)	1 (1.9)	1.000
3	51 (98.1)	51 (98.1)	
**Laboratory results on admission**			
Troponin-I, ng/mL	25.3 (4.9, 46.7)	40.9 (14.2, 76.6)	0.021
BNP, ng/L	80.3 (45.4, 213.3)	169.0 (61.8, 277.3)	0.030
Total cholesterol, mmol/L	4.5 (3.9, 5.3)	5.0 (4.4, 5.7)	0.023
LDL, mmol/L	2.8 ± 0.8	3.3 ± 1.1	0.006
HDL, mmol/L	0.9 (0.8, 1.1)	1.0 (0.8, 1.2)	0.148
Triglycerides, mmol/L	1.5 (1.0, 2.4)	1.4 (0.9, 2.2)	0.528
**Concomitant medications**			
Aspirin	52 (100.0)	52 (100.0)	1.000
Clopidogrel	28 (53.8)	26 (50.0)	0.695
Ticagrelor	24 (46.2)	26 (50.0)	0.695
β-blocker	35 (67.3)	36 (69.2)	0.833
ACE-I/ARB	31 (59.6)	35 (67.3)	0.415
Statin	52 (100.0)	52 (100.0)	1.000

Data are presented as n (%), median (IQR), or mean ± SD.

MSI, myocardial salvage index; TIMI, thrombolysis in myocardial infarction; PCI, percutaneous coronary intervention; BNP, brain natriuretic peptide; LDL, low-density lipoprotein; HDL, high-density lipoprotein; ACE-I, angiotensin-converting enzyme inhibitor; ARB, angiotensin receptor blocker.

**TABLE 2:** Cardiovascular magnetic resonance results.

	MSI ≥ Median MSI (*n* = 52)	MSI < Median MSI (*n* = 52)	*P*-value
LVEDV, mL/m^2^	134.6 ± 26.7	132.8 ± 28.0	0.738
LVESV, mL/m^2^	64.3 (50.5, 78.9)	72.4 (54.9, 87.4)	0.118
LVEF,%	51.3 ± 11.4	45.3 ± 12.1	0.011
IS,% LV	8.7 (6.0, 11.9)	20.4 (14.9, 25.0)	<0.001
AAR,% LV	38.2 (31.6, 41.9)	33.3 (24.0, 39.2)	0.020
MVO	19 (36.5)	31 (59.6)	0.019
MVO, % LV	0.0 (0.0, 1.1)	0.4 (0.0, 2.4)	0.013

Data are presented as n (%), median (IQR), or mean ± SD.

MSI, myocardial salvage index; LVEDV, left ventricular end-diastolic volume; LVESV, left ventricular end-systolic volume; LEVF, left ventricular ejection fraction; IS, infarction size; LV, left ventricle; AAR, area at risk; MVO, microvascular obstruction.

## Results

**FIGURE 1:**
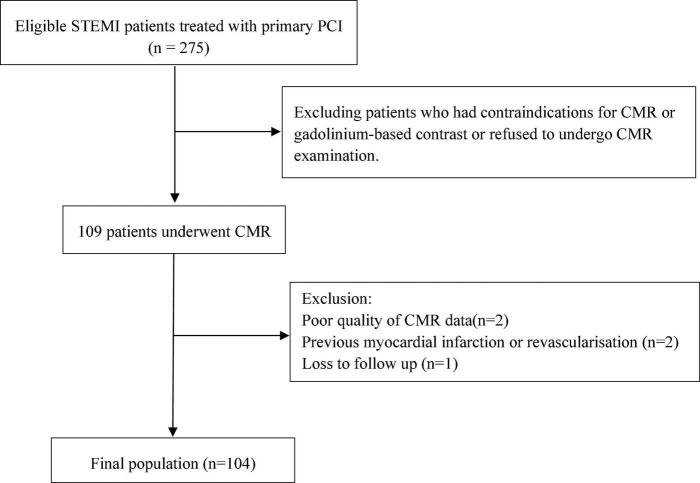
Study flow chart. STEMI, ST-segment elevation myocardial infarction; PCI, percutaneous coronary intervention; CMR, cardiovascular magnetic resonance.

**FIGURE 2:**
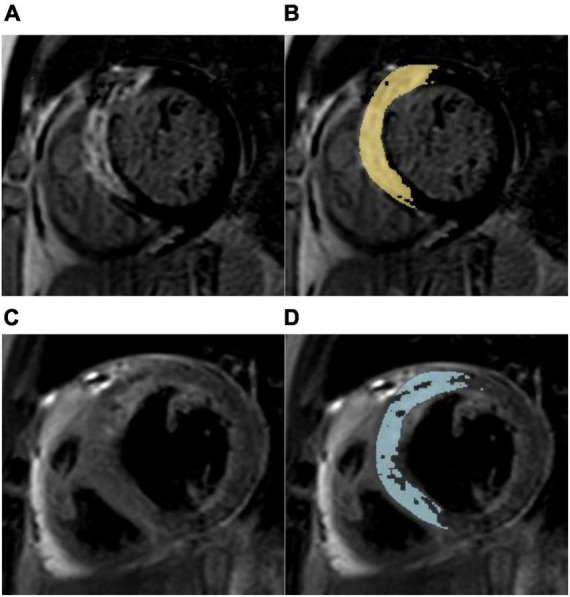
Myocardial salvage assessment in a 67-year-old male with a myocardial salvage index < median after acute reperfused myocardial infarction. **(A)** Representative short-axis late gadolinium enhancement (LGE) image showing a high signal intensity of the anterior, anteroseptal, and inferoseptal segments indicating myocardial necrosis. **(B)** Computer-aided signal intensity analysis of LGE image normalized to remote uninjured myocardium. The myocardial infarct size was 20.95%LV (left ventricle). **(C)** T2-weighted spectral attenuated inversion recovery image showing myocardial edema (area at risk) in the same region. **(D)** Computer-aided signal intensity analysis of the T2-weighted image normalized to remote normal myocardium. The myocardial area at risk was 34.30% LV, and the myocardial salvage index was 39.1. This patient with anterior myocardial infarction suffered non-fatal myocardial reinfarction and congestive heart failure during a 3-year follow-up.

This prospective study included 104 patients [mean age 56.3 years, 85 (81.7%) males] with first-time STEMI who were examined by CMR imaging 4 days (IQR 3–6) after P-PCI. The study flow chart is illustrated in Figure 1. According to the median MSI (54.5), 104 patients were divided into two groups with 52 patients in each group (Figure 2).

### Baseline characteristics of the patients

An overview of patients′ baseline characteristics, angiographic findings, laboratory results, and concomitant medications is presented in Table 1. Patients in the MSI ≥ median group were more likely to be male sex, as shown in Table 1 (*P* = 0.022). The concomitant medications of patients were similar across the groups. In terms of laboratory results on admission, patients with MSI < median had higher baseline levels of troponin-I, brain natriuretic peptide (BNP), total cholesterol, and low-density lipoprotein, as shown in Table 1 (all *P* < 0.05). There was no significant difference in other clinical characteristics between the two groups (all *P* > 0.05).

### Cardiovascular magnetic resonance results

Patients underwent CMR at 4 days (IQR 3–6) after P-PCI. The major CMR results are shown in Table 2, the median AAR was 34.7% LV (IQR 27.1–41.4), and the median IS was 13.5% LV (IQR 8.6–20.4) in this study. The median MSI calculated from these was 54.5 (IQR 39.6–75.3). On initial CMR scans, MVO was identified in 50 (48.1%) patients, and the incidence of MVO was significantly higher in patients with MSI < median group [31 (59.6%) vs. 19 (36.5%), *P* = 0.019]. Compared with the MSI ≥ median group, IS and the extent of MVO in the < median MSI group were significantly larger, while LVEF was significantly smaller as shown in Table 2 (all *P* < 0.05).

### Intra- and interobserver variability

Intra- and interobserver agreements were excellent for IS (ICC 0.920 and 0.938, respectively), and good to excellent for AAR (ICC 0.894 and 0.918, respectively). Bland-Altman plots were shown in Supplementary material. The Bland-Altman plots used to assess limits of agreement and bias gave satisfactory results (Supplementary material).

### Clinical outcome

**FIGURE 3:**
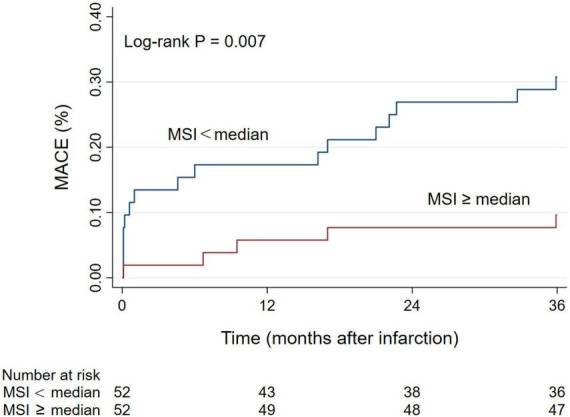
Kaplan-Meier curve of the cumulative incidence of MACE during 3-year follow-up. MACE, major adverse cardiac events; MSI, myocardial salvage index.

During the 3-year follow-up period, 21 patients (20.2%) experienced a MACE, of which 4 patients (3.8%) died of all causes, 8 patients (7.7%) developed non-fatal myocardial reinfarction, and 11 patients (10.6%) had new congestive heart failure. Patients in the MSI < median group had a higher incidence of MACE [16 (30.8%) vs. 5 (9.6%), *P* = 0.007] and congestive heart failure [9 (17.3%) vs. 2 (3.8%), *P* = 0.026]. The Kaplan-Meier cumulative incidence curve of MACE is provided in Figure 3. We assessed the association between MSI and clinical outcomes and calculated HR (95% CI) using the Cox analysis in Table 3. In univariate Cox analysis, Killip class on admission, anterior myocardial infarction, door-to-balloon time, the levels of troponin-I, BNP, and LVEF, the presence of MVO, the extent of MVO, IS, and MSI were associated with the increased MACE risk (all *P* < 0.05). After stepwise multivariate Cox regression analysis, MSI (HR 0.963, 95% CI: 0.943–0.983; *P* < 0.001) together with the level of BNP (HR 1.002, 95% CI: 1.001–1.003; *P* = 0.001) remained independently significant as predictors of MACE (Table 3). In the secondary endpoint analysis, we found that MSI (HR 0.967, 95% CI: 0.938–0.997; *P* = 0.032) was an independent predictor of congestive heart failure through stepwise multivariate Cox regression analysis (Table 3).

**TABLE 3:** Predictors of MACE and congestive heart failure in univariable and stepwise multivariable Cox regression analysis.

	Univariable		Stepwise multivariable	
		
	Hazard ratio (95% CI)	*P*-value	Hazard ratio (95% CI)	*P*-value
**MACE**				
Killip class on admission	1.891 (1.089, 3.284)	0.024	–	–
Anterior myocardial infarction	3.939 (1.527, 10.159)	0.005	–	–
Door-to-balloon time	1.005 (1.002, 1.009)	0.004		
Troponin-I	1.021 (1.005, 1.037)	0.009	–	–
BNP	1.002 (1.001, 1.003)	0.001	1.002 (1.001, 1.003)	0.001
LVEF	0.965 (0.933, 0.999)	0.043	–	–
MVO	3.010 (1.167, 7.763)	0.023	–	–
MVO (% LV)	1.197 (1.038, 1.380)	0.013	–	–
IS (% LV)	1.044 (1.011, 1.078)	0.009	–	–
MSI	0.962 (0.941, 0.984)	0.001	0.963 (0.943, 0.983)	<0.001
**Congestive heart failure**				
Anterior myocardial infarction	6.734 (1.453, 31.204)	0.015	5.173 (1.094, 24.465)	0.038
Troponin-I	1.024 (1.001, 1.046)	0.037	–	–
MSI	0.960 (0.931, 0.990)	0.010	0.967 (0.938, 0.997)	0.032

MACE, major adverse cardiac events; BNP, brain natriuretic peptide; MVO, microvascular obstruction; LV, left ventricle; IS, infarction size; MSI, myocardial salvage index.

### Comparison of myocardial salvage index and global registry of acute coronary events risk scores in predicting prognosis

**FIGURE 4:**
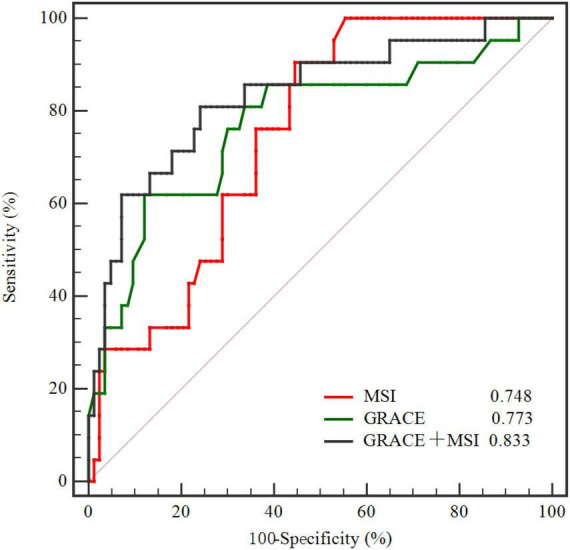
Comparison of ROC curves demonstrating discrimination of the MSI, GRACE, and GRACE + MSI in predicting MACE. MSI, myocardial salvage index; GRACE, Global Registry of Acute Coronary Events; ROC, receiver operating characteristic; MACE, major adverse cardiac events.

ROC curve illustrated that MSI [AUC, 0.748 (95% CI: 0.654–0.828)] was a strong indicator of MACE in 3-year follow-up (Figure 4), and the best cut-off value of MSI for predicting MACEs was 57.5 with a sensitivity of 90.48% and a specificity of 55.42%. The discriminatory performance of MSI was similar to the GRACE risk score [AUC, 0.773 (95% CI: 0.680–0.849)] in predicting MACEs (*P* = 0.756). However, adding MSI to the GRACE risk score [AUC, 0.833 (95% CI: 0.747–0.899)], the discriminatory performance significantly increased (*P* = 0.044) compared with the GRACE risk score (Figure 4 and Table 4). Meanwhile, it had a better prognostic performance than the GRACE risk score alone in predicting MACE during a 3-year follow-up period with an NRI of 0.635 (*P* = 0.009) and an IDI of 0.101 (*P* = 0.002) (Table 4).

**TABLE 4:** Model performance after the addition of MSI to the GRACE risk score.

	AUC (95% CI)	*P*-value	NRI	*P*-value	IDI	*P*-value
GRACE risk score	0.773 (0.680, 0.849)	Ref	Ref	Ref	Ref	Ref
GRACE risk score + MSI	0.833 (0.747, 0.899)	0.044	0.635	0.009	0.101	0.002

MSI, myocardial salvage index; GRACE, Global Registry of Acute Coronary Events; AUC, area under the curve; NRI, net reclassification improvement; IDI, integrated discrimination improvement.

## Discussion

This prospective study investigated the prognostic value of MSI as assessed by CMR for 3-year hard clinical outcomes in 104 first-time STEMI patients treated with P-PCI. Our findings demonstrated that: (a) patients in the MSI < median group had a significantly higher incidence of MACE compared with the MSI ≥ median group, and MSI was an independent predictor of MACE and congestive heart failure in patients with STEMI after the P-PCI treatment in a 3-year follow-up; (b) the discriminatory performance of MSI was close to the GRACE risk score, and adding MSI to the GRACE risk score could significantly improve the ability of the GRACE risk score in predicting the risk of incident MACE.

Eitel et al. (23) demonstrated for the first time that MSI assessed by CMR can independently predict the short-term prognosis of acute STEMI after the P-PCI treatment through the analysis of 208 patients with first-time STEMI and a 6-month follow-up study. About a year later, the same team published a study on the prediction of long-term clinical endpoints by MSI, in which 202 patients with STEMI in the same population were followed up for a median of 18.5 months (13). They found that MSI assessed by CMR could independently predict the long-term cardiac death after the P-PCI treatment in STEMI patients and that MSI had prognostic value. Both Eitel and our studies confirmed the value of MSI assessed by CMR in predicting the long-term prognosis in acute reperfused STEMI, except that our results also demonstrated that MSI assessed by CMR was associated with congestive heart failure. Masci et al. (12) found that CMR-derived MSI was a strong independent predictor of adverse LV remodeling in reperfused STEMI patients, which remained unchanged after adjusting important parameters (MVO, MI transmurality, and baseline LVEF) in multivariate analysis. LV remodeling is an important pathophysiological process of various types of heart disease (24). Ventricular remodeling refers to the process of scar enlargement in a large infarcted area, followed by local ventricular dilatation and functional changes. Postinfarction remodeling has the highest risk of symptomatic heart failure, which is related to adverse clinical outcomes (25). It might explain the association between MSI and congestive heart failure in this study.

From a pathological point of view, cell injury can be induced in the early stage after coronary artery occlusion (26). Irreversible myocardial injury can be caused by persistent occlusion that first involves the subendocardium and then extends to the subepicardium as a wavefront (27). Timely and effective reperfusion can limit IS, save ischemic myocardium and reduce ischemic myocardial necrosis (26). Therefore, to evaluate the effectiveness of reperfusion therapy, determining the number of cardiomyocytes saved by measuring IS and AAR was warranted. IS influenced by the size of AAR, residual blood flow to the region of ischemia, metabolic demands of the myocardium, and the duration of coronary occlusion, and the minor difference in AAR may lead to significant changes in IS (12). While IS provides a rough estimate of myocardial salvage, the extent of AAR is high-variability and relies on the IRA, distribution of the coronary artery, and location of culprit lesion (28). Theoretically, the normalization of IS by AAR can decrease the variability in measurements. Therefore, compared with absolute IS, the proportion of AAR would be a better indicator to measure the effect of treatment (28). MSI is calculated by adjusting for the amount of myocardial necrosis within the range of AAR, and its correlation to prognosis might be helpful to select patients who may benefit from drug or device treatment.

Early risk stratification is the key to identify high-risk patients to further guide clinical treatment and improve patient outcomes. The GRACE risk score is now widely recommended for risk stratification and prognosis prediction in ACS (18), including the major traditional clinical risk factors. However, the prognosis of patients with STEMI is affected by many factors, and, more and more new risk factors have been found and studied, which are not included in the GRACE risk score in recent years (29–31). As the gold-standard technique for the characterization of cardiac structure and function, CMR has been developed as a unique tool for assessing post-myocardial infarction over the past 20 years and has shown the prospect of improving the risk stratification in STEMI patients (32). Our study found that adding MSI assessed by CMR to the GRACE risk score could significantly increase the discriminatory performance and produce a stronger predictive value. As one of the imaging metrics, CMR-derived MSI is independently associated with adverse LV remodeling (12), which accounts for the adverse clinical outcomes of STEMI patients from the perspective of imaging. Both GRACE risk score and MSI reflect somewhat different risk attributes in predicting adverse outcomes, and their synergistic use can enhance risk stratification to comprehensive assess clinical prognosis. Although CMR has advanced our understanding about the acute phase of reperfused STEMI, it is currently not feasible to add CMR as a routine clinical examination because of its high cost and inconvenience.

Some limitations need attention. First, this is a single-center study, so the number of patient samples is limited. Large sample, multi-center studies are needed to further confirm our findings. Second, CMR was detected as markers of myocardial reperfusion after myocardial infarction, but other methods, such as ST-segment resolution, were not explored in this study. Third, the new CMR T1, T2 mapping and dark blood techniques not used in the study are worthy of further study in the assessment of myocardial damage. The former provides a quantitative assessment of myocardial damage (33), and dark blood LGE can improve the diagnostic accuracy of myocardial infarction, including reducing missed diagnosis and misdiagnosis caused by the bright edge of blood pool or subendocardial trabecular hemorrhage (34, 35).

## Conclusion

Our study confirmed that MSI assessed by CMR was an independent predictor of MACE in first-time STEMI patients treated with P-PCI. It also could additionally improve the prognostic performance of the GRACE risk score and providing significant incremental value in clinical risk stratification for STEMI patients.

## Data availability statement

The datasets presented in this article are not readily available because the privacy of patients or ethical restrictions. Requests to access the datasets should be directed to corresponding author.

## Ethics statement

The studies involving human participants were reviewed and approved by Shengjing Hospital of China Medical University Ethics Committee. Written informed consent for participation was not required for this study in accordance with the national legislation and the institutional requirements.

## Author contributions

SZ wrote the manuscript. ZQS and LZ conceived and designed the study. QM, TY, YH, YJ, JW, and ZJS participated in data collection. SZ and LZ performed the statistical analysis. All authors contributed to the article and approved the submitted version.
