# Supplementary material for: Prognostic value of myocardial salvage index assessed by cardiovascular magnetic resonance in reperfused ST-segment elevation myocardial infarction
Source: Front Cardiovasc Med. 2022 Aug 16;9:933733. doi: 10.3389/fcvm.2022.933733 (PMC9425200; doi:10.3389/fcvm.2022.933733)
Supplement: Supplementary file 1 [file Table_1.pdf]

Supplementary Material

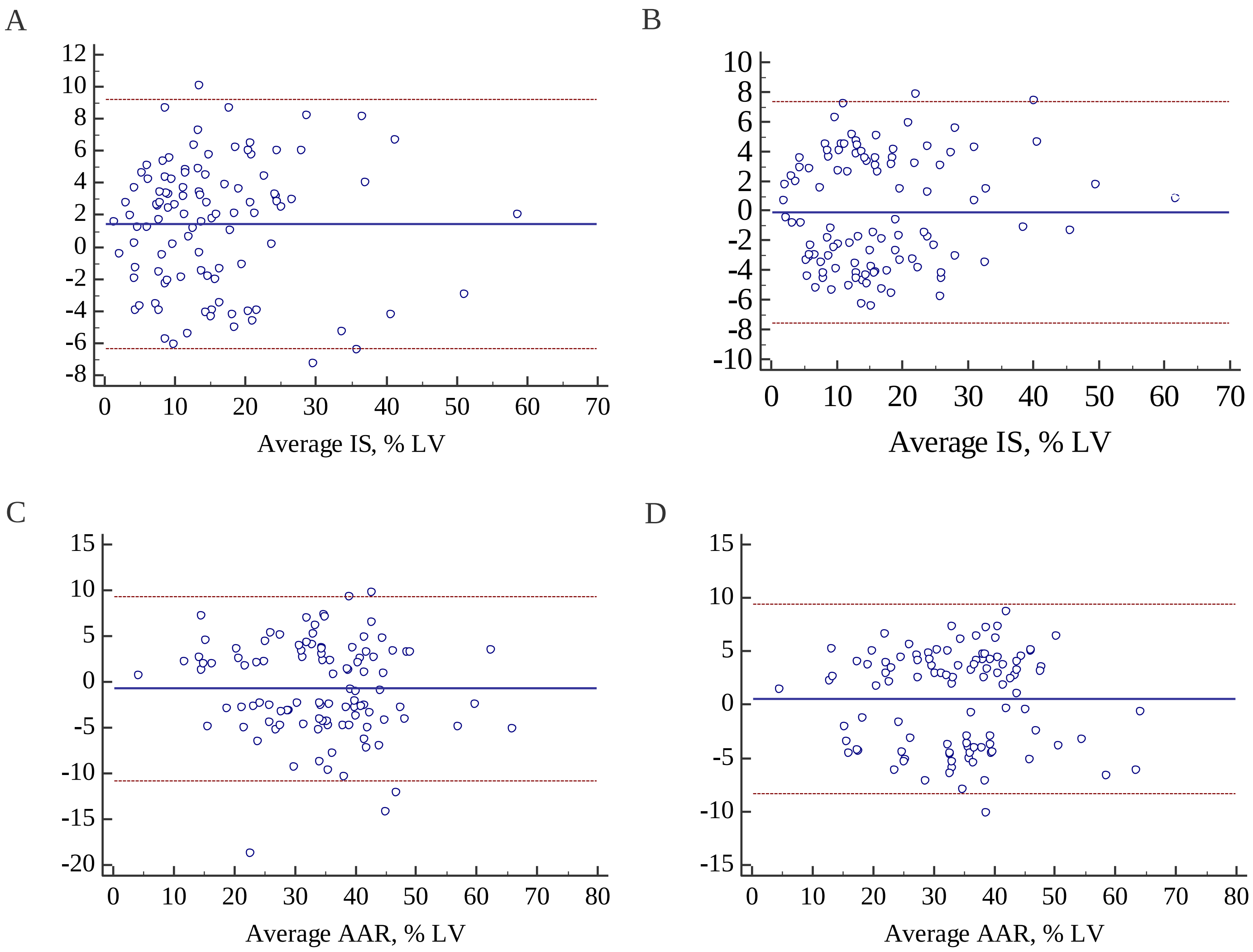

Supplementary Figure 1: Bland-Altman plots show agreement for A: reviewer 1 and reviewer 2 for the determination of IS; B: reviewer 1 for the determination of IS; C: reviewer 1 and reviewer 2 for the determination of AAR; D: reviewer 1 for the determination of AAR.
